# Effect of Niobium Content on the Microstructure and Mechanical Properties of Simulated Coarse-Grained Heat-Affected Zone (CGHAZ) of High-Strength Low-Alloy (HSLA) Steels

**DOI:** 10.3390/ma15093318

**Published:** 2022-05-05

**Authors:** Hongwei Yu, Kaiming Wu, Baoqi Dong, Liling Yu, Jingxi Liu, Zicheng Liu, Daheng Xiao, Xing Jing, Hankun Liu

**Affiliations:** 1 The State Key Laboratory of Refractories and Metallurgy, Wuhan University of Science and Technology, Wuhan 430081, China; yuhongwei@baowugroup.com (H.Y.); dongbq2016@163.com (B.D.); yuliling2022@163.com (L.Y.); 2 School of Naval Architecture and Ocean Engineering, Huazhong University of Science and Technology, Wuhan 430074, China; liu_jing_xi@hust.edu.cn; 3 Department of Manufacturing, Baoshan Iron & Steel Co., Ltd., Shanghai 201999, China; liuzc@baosteel.com; 4 Technology Center, Hunan Valin Xiangtan Steel Co., Ltd., Xiangtan 411101, China; xiaodh123@126.com; 5 Department of Manufacturing, Nanjing Iron & Steel Co., Ltd., Nanjing 210044, China; jinxing@njsteel.com.cn; 6 R&D Center, China Petroleum Group Ocean Engineering (Qingdao) Co., Ltd., Qingdao 266520, China; liuhk2022@163.com

**Keywords:** coarse-grained heat-affected zone (CGHAZ), martensite/austenite constituent (M/A), welding thermal cycle, impact toughness, niobium microalloying

## Abstract

The effect of Nb-content and heat input rate on the mechanical properties and microstructure of simulated coarse-grained heat-affected zone (CGHAZ) of high-strength low-alloy steel (HSLA) was investigated. While using a low heat input (20 kJ/cm), the toughness of simulated CGHAZ was improved by increasing the Nb-content. The maximum toughness was obtained when the Nb-content was 0.110 wt.% and the heat input was 20 kJ/cm. The samples made at this condition had fine martensite/austenite (M/A-constituent), acicular ferrite and refined austenite grains. As the heat input was increased to 200 kJ/cm, the toughness of simulated CGHAZ was significantly decreased irrespective of the Nb-content because of the formation of coarse austenite grains, low angle grain boundaries, and massive M/A-constituents.

## 1. Introduction

Engineering steels are attractive for structural applications because of their high strength and toughness at relatively low costs [1]. They are widely used for the construction of bridges, pipelines, railways, and automobiles, with service temperatures from subzero up to ~600 °C.

The use of steels generally involves welding, a process that is governed by various metallurgical factors. Given that the application of steels is being extended to extreme environments such as those witnessed in the North Sea and the Gulf of Alaska for exploration of oils and gas, higher toughness of weldments is an important requirement [2]. However, achieving high toughness in steels with the yield strengths above 700 MPa is a challenging issue [3].

The use of Nb in steels as a microalloying element started around the 1970s. It has attracted significant attention due to its strengthening and toughening effects on HSLA steels, especially those produced by TMCP (thermo-mechanical controlled processing). Several studies have indicated that the addition of a small amount of Nb results in the formation of carbonitride, which refines austenite grains and thereby effectively improves both strength and toughness [4,5,6]. Recent studies indicated that Nb not only increases the mechanical properties, but also improves the corrosion resistance in sea water and atmosphere [7,8,9,10,11,12,13]. Although the microalloying of Nb is helpful to achieve excellent toughness by means of TMCP in base metal, the role of the microalloying element on toughness of the heat-affected zone (HAZ), especially CGHAZ at high heat input, is not well understood.

Niobium is reported to weaken HAZ toughness because of martensite hardening, even though it improves the toughness and increases the strength of the base plate [14,15]. Niobium also limits the ferrite nucleation on TiN by producing unstable carbonitride, and promotes the formation of coarse upper bainite at extended cooling times (*t*_8/5_ ≥ 50 s) [16,17]. Some studies have also indicated the micro-segregation of manganese and niobium in high carbon steels. Meanwhile, Nb increases the hardenability of re-austenitized structure during the second thermal cycle, helping to form the M/A constituent. Moreover, in certain conditions, it retards the decomposition of M/A by preventing carbon diffusion [18,19].

The impact of Nb on HAZ toughness depends significantly on the contents of carbon and Nb. In the high content of niobium and carbon steels, the micro-segregation of Nb and Mn, and the carbon segregation in un-transformed austenite near grain boundaries, may attribute to the formation of the M/A constituent [20,21]. Low Nb-content i.e., ≤0.02%, is suggested to improve the heat-affected zone toughness in TMCP and normalized steel with yield strength ≥ 355 MPa. It also reported that the Nb content ≥ 0.04% can be utilized without any detrimental effect on the toughness of HAZ, if the carbon content of steel is kept below 0.03–0.04 wt.% in X80 pipeline steels [14]. Therefore, the effects of different amounts of Nb on base metals and HAZ have been extensively investigated [22,23]. However, limited investigation has been carried out on the effect of high amounts of Nb on the microstructure and mechanical properties of HAZ, especially CGHAZ.

The growing interest in welding technology with high heat input and more stricter requirements for fracture toughness in structural applications has led to the demand of superior CGHAZ toughness. Currently, the effect of high Nb-content during high input welding on the microstructure and mechanical properties of CGHAZ is not well understood, which is the underlying reason for this study. The role of grain size, grain boundary misorientation, and the M/A constituent on simulated CGHAZ in microalloyed steels with higher Nb-content was studied in this work, aiming to provide generic guidelines in the design and welding of Nb-microalloyed HSLA steels.

## 2. Materials and Methods

The HSLA steels were industrially produced via conventional continuous casting, TMCP, followed by a tempering process. The steel samples were remelted with two amounts of Nb-content (0.073%, 0.110%) in a vacuum smelting furnace and then cast into ingots. The fully solidified ingots were forged into 15 mm plates in a pilot plant. The size of all specimens was 11 × 11 × 55 mm^3^.

The chemical compositions of tested steels are listed in Table 1. All concentrations are given in wt.%.

The heat input of 20 kJ/cm is usually used in industrial welding, for example in submerged arc welding and gas shielding welding (CO_2_ + Ar). The heat input of 200 kJ/cm is more common in electro-gas welding. Therefore, 20 kJ/cm is selected to simulate submerged arc welding and gas shielding welding, whereas 200 kJ/cm was meant to simulate electro-gas welding. In order to obtain simulated CGHAZ with different heat inputs (E) of 20 and 200 kJ/cm, experiments were carried out using a Gleeble 3800 machine (Dynamic System Inc., Poestenkill, NY, USA) for different cooling rate of *t*_8/5_ (i.e., from 800 to 500 °C). The size of the specimen was 11 × 11 × 55 mm^3^.

The relation between heat input *E* and cooling time *t*_8/5_ (from 800 to 500 °C) is given in Equation (1).

(1) $$t_{8/5}=(0.67-5\times 10^{-4}T_{0})E(\frac{1}{500-T_{0}}-\frac{1}{800-T_{0}})$$

where *T*_0_ is the initial temperature (20 °C). The cooling times (from 800 to 500 °C) were 10.6 s, and 105.6 s respectively, which corresponded approximately with the welding heat input of 20 and 200 kJ/cm.

**Figure 1:**
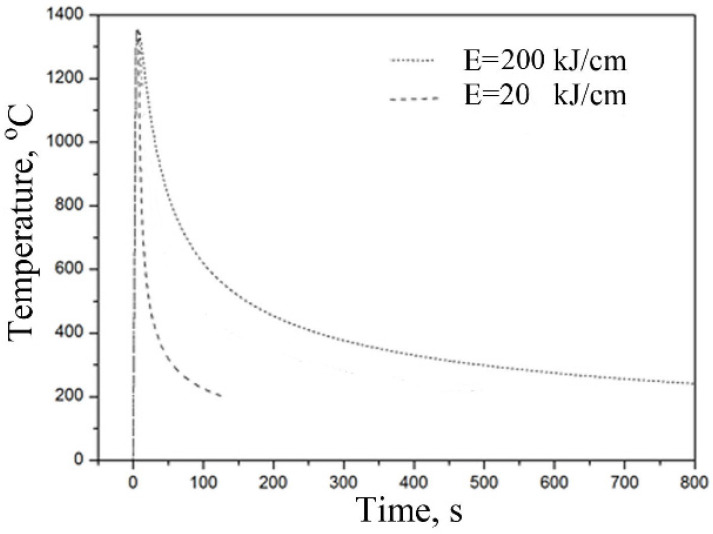
Thermal cycles used in the simulation of CGHAZ.

There are four parameters that influence simulation: heating rate from initial temperature to peak temperature (*R_h_*), peak temperature (*T_p_*), holding time at peak temperature (*t_h_*) and cooling time of *t*_8/5_. Table 2 shows the simulation parameters. The thermal cycles for simulation of HAZ are presented in Figure 1.

The test samples were polished by standard metallographic methods and etched with 4 vol.% nital solution prior to optical and scanning electronic microscopy. To reveal the M/A constituent, polished specimens were initially electro-etched for 10 s at 3 V in a solution consisting of a mixture of 5 g tetra-acetic acid (EDTA) and 0.5 g NaF in 100 mL distilled water. In the second stage they were elector-etched for 60 s at 6 V in a solution consisting of a mixture of 5 g picric acid and 25 g NaOH in 100 mL distilled water.

The detailed observation of the microstructure was done by scanning electron microscope (SEM). Semi-automatic electrolytic polishing etching equipment was used to electrolytically polish the pre-polished specimens. EBSD (electron backscatter diffraction) was used for the analysis of the grain boundary misorientation and crystallographic grain size.

The impact toughness tests on standard V-notched Charpy specimens were conducted at −20 °C.

## 3. Results

### 3.1. Microstructural Analysis

**Figure 2:**
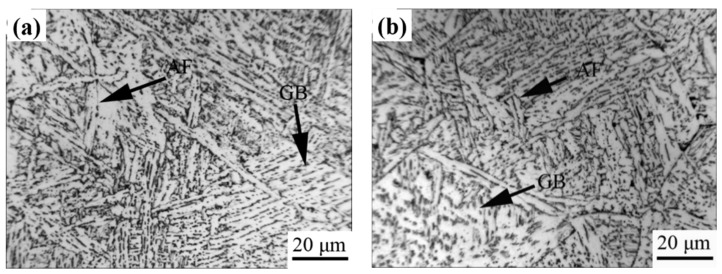
Optical structure of simulated CGHAZ with a heat input of 20 kJ/cm in the steel samples containing different amounts of. (**a**) 0.073%, (**b**) 0.110%.

Figure 2 shows optical microstructures of the simulated CGHAZ in the steel samples containing 0.073% and 0.110% Nb when the heat input was 20 kJ/cm. The microstructure of CGHAZ for 0.073 wt.% Nb steel mainly consisted of bainitic packets and acicular ferrite laths or plates (Figure 2a). When the content of Nb increased up to 0.110 wt.%, the microstructure of CGHAZ was predominantly acicular ferrite along with granular bainite. The acicular ferrites were long and coarse (Figure 2b). It was also noted that the prior austenite grains became coarser with increasing Nb-content (Table 3). The average austenite grain size was about 34 μm in the 0.073% Nb and 46 μm in 0.110% Nb samples.

**Figure 3:**
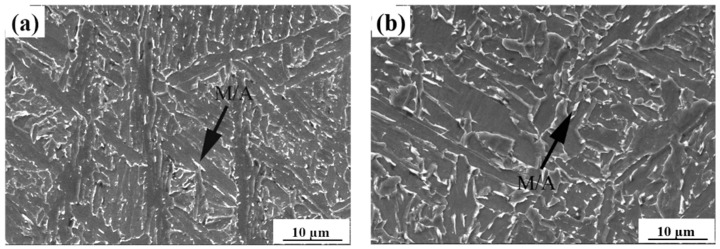
SEM images of simulated CGHAZ with a heat input of 20 kJ/cm in the steels containing. (**a**) 0.073%, (**b**) 0.110% Nb.

Figure 3 shows scanning electron micrographs in the specimens with a heat input of 20 kJ/cm. When the heat input was so low, the majority of martensite/austenite (M/A constituent) had a fine structure, which is good for impact toughness.

**Figure 4:**
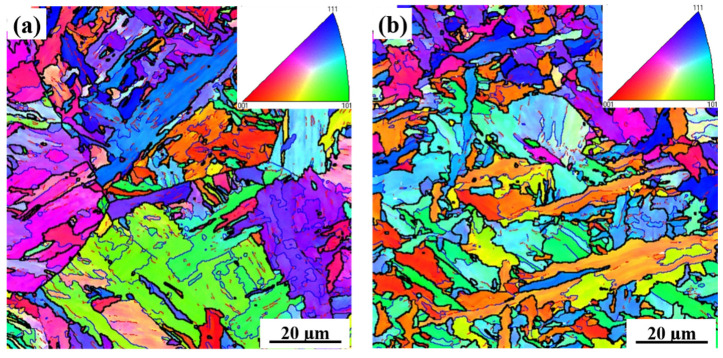
Orientation maps of bcc-phase in normal direction simulated CGHAZ with a heat input of 20 kJ/cm in the steel samples containing (**a**) 0.073% and (**b**) 0.110% Nb.

Figure 4 shows bcc-phase orientation maps in the steel containing different amounts of Nb. As seen from Figure 4 and Table 3, the crystallographic grains became smaller as the Nb content was increased. Table 4 provides the analysis of grain boundary misorientation angle between neighboring grains. It is clear that a considerably higher fraction of the high-angle grain boundary (>10°) was present in the specimens containing 0.110% Nb compared to that of 0.073% Nb samples.

**Figure 5:**
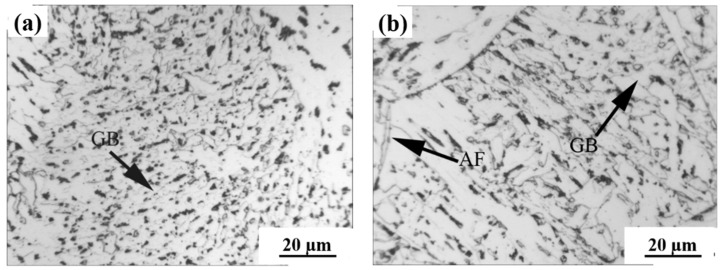
Optical micrographs of simulated CGHAZ with a heat input of 200 kJ/cm with (**a**) 0.073%, (**b**) 0.110% Nb.

Figure 5 shows that with a high heat input, the microstructure of CGHAZ for 0.073% Nb was predominantly granular bainite, whereas the sample with 0.110% Nb contained some acicular ferrite. Comparing the data given in Table 3 and Table 5, the prior austenite grains were larger when the heat input was higher in the samples with the same amount of Nb.

**Figure 6:**
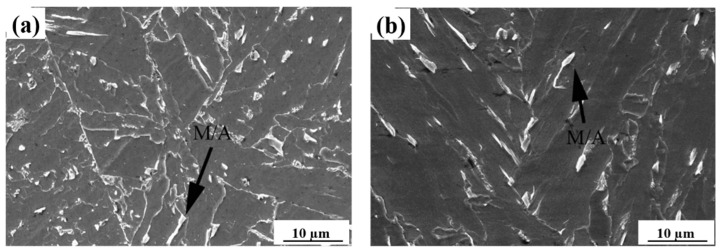
SEM images of simulated CGHAZ with a heat input of 200 kJ/cm with (**a**) 0.073% Nb and (**b**) 0.110% Nb.

Figure 6 shows the specimen containing 0.073 wt.% Nb had elongated M/A constituents, mostly precipitated at the grain boundaries. In contrast, most of M/A constituents were elongated in the 0.110% Nb samples.

**Figure 7:**
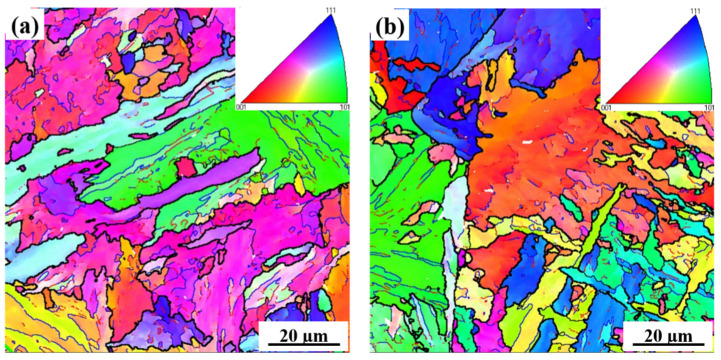
Orientation image maps of microstructure in normal direction in samples simulated with a heat input of 200 kJ/cm with (**a**) 0.073% and (**b**) 0.110% Nb.

Figure 7 shows the size of crystallographic grains in the simulated CGHAZ samples listed in Table 5. It is clear that the austenite grains became larger as the Nb content was increased. Table 6 provides the analysis of grain boundary misorientation angle between the neighboring grains shown in Figure 7. Clearly the population of the high-angle grain boundaries (>10°) was increased with the increase of Nb-content.

### 3.2. Impact Toughness Tests

Table 7 shows the results of impact tests carried out on the simulated CGHAZ samples at −20 °C. It is clear that, regardless of Nb content, generally the impact toughness was low when the heat input was high and it was worsened by increasing Nb content. Interestingly and in contrast, the impact toughness was increased with increasing Nb content if the heat input was low.

## 4. Discussion

Most welded plain carbon steels suffer from grain coarsening. The addition of microalloying elements, such as Nb, Ti and V, can effectively reduce coarsening, which is widely used on an industrial scale [14,15,16,24,25,26]. The addition of Nb and Ti facilitate the formation of carbonitride particles, which can pin the austenite grain boundary and retard the coarsening of the heat-affected zone. However, the addition of too much Nb (e.g., 0.110%) resulted in coarsening, the size of carbonitride precipitates was increased [9,16], and hence the observed reduced pinning effect, as shown in Table 3.

Acicular ferrite can increase both the toughness and strength of steels because of their shape and properties. Acicular ferrite prefers to nucleate on inclusions under a suitable cooling rate in heat-affected zone of HSLA steels [27,28]. It has been confirmed that oxide and sulfide inclusions can significantly promote the formation of acicular ferrite [29,30,31,32]. Nitrides and carbonitrides formed by microalloying elements such as Ti, V and Nb can also be beneficial for the formation of acicular ferrite [33,34]. Besides chemical composition, the size of austenite grains can also influence the formation of acicular ferrite. For instance, Nb can promote the precipitation of carbonitrides, which provide more nucleation sites for acicular ferrite [9,16]. Meanwhile because of reduction in TiN, an effective pinning element, the austenite grains are coarsened, and coarse austenite grains provide more space for nucleation of acicular ferrite. It was reported that the optimum size of acicular ferrite is between 50 to 110 μm [35,36].

The M/A constituent is a key phase transformed in the CGHAZ of HSLA steels and can significantly influence the toughness of weldment. Predominantly, the formation of M/A constituents depends on the steel’s chemical composition and the cooling rate [37,38,39]. During the phase transformation from austenite to bainite or ferrite, carbon atoms are continuously expelled into γ phase. Such uneven carbon distribution within the untransformed austenite improves the stability [40]. During welding with a low heat input, fine M/A constituents were formed in the simulated CGHAZ containing 0.073% Nb samples. The M/A constituents became finer in the samples with higher Nb content (i.e., 0.110 wt.%), M/A constituents became finer and the amount increased with increasing Nb content (Figure 3). This is because increasing the Nb content promotes the micro-segregation of niobium and manganese in the regions enriched with carbon, and thus increases the M/A constituent amount. Meanwhile, Nb retards M/A decomposition by means of preventing the diffusion of carbon atoms during thermal cycles [18,20]. Such a fine and film-like M/A constituent can change the direction of cracking and retard dislocation movement and also limit crack propagation [21,41]. Therefore, the addition of Nb is beneficial for strength and toughness improvement provided the heat input remains low.

In contrast, the high heat input welding processes lead to the formation of massive M/A constituents that are formed and distributed at grain boundaries, which are deleterious to toughness owing to the fact that they can act as nucleation sites for cracking. This is the main reason behind the observed low toughness when a high heat input was applied.

From Table 4, it can be seen that the higher the Nb content, the more the high-angle grain boundaries, and consequently the higher the toughness. The addition of Nb can promote the formation of carbonitride precipitates, which are suitable nucleation of acicular ferrite [9,16], and this leads to more high-angle grain boundaries [42,43,44]. The high-angle grain boundary can change the cracking direction and effectively obstruct cracking propagation [6,42,43].

## 5. Conclusions

(1) By applying a low heat input (20 kJ/cm), the measured V-notch impact toughness of simulated CGHAZ was increased with the increase of Nb-content. This was attributed to the formation of acicular ferrite and more homogeneous distribution of fine M/A constituents in high Nb-bearing steel. Meanwhile, acicular ferrite could refine austenite grains with many high angle grain boundaries, which inhibited the propagation of cracks, hence the observed improvement in toughness.
(2) By applying a high heat input (200 kJ/cm), coarse austenite grains were formed in the simulated CGHAZ, and impact toughness was significantly dropped regardless of the Nb-content.

## Figures and Tables

**Table 1:** Chemical analysis of tested steels (wt.%).

No.	C	Mn	Si	V	Nb	Ti	Al	Fe
(a)	0.062	1.55	0.20	0.021	0.073	0.027	0.013	Balance
(b)	0.052	1.62	0.21	0.022	0.110	0.020	0.012	Balance

**Table 2:** Welding simulation parameters.

E	*R_h_*	*T_p_*	*t_h_*	*t*_8/5_
20 kJ/cm	300 °C/s	1350 °C	3 s	10.6 s
200 kJ/cm	300 °C/s	1350 °C	3 s	105.6 s

**Table 3:** Measured austensite grain size in simulated CGHAZ.

No.	Size of Austenite Grains (μm)	Size of Crystallographic Grains (µm)
(a)	34.75	6.51
(b)	45.91	6.37

**Table 4:** Percentage of different grain misorientation angles corresponding to samples shown in Figure 4.

Sample	<3° (%)	>5° (%)	>10° (%)	>15° (%)	>30° (%)	>40° (%)
(a)	60.19	30.94	19.83	15.85	14.36	14.01
(b)	49.35	45.21	33.93	29.69	27.75	27.61

**Table 5:** Measured austensite grain size in simulated CGHAZ corresponding to samples shown in Figure 5.

No.	Size of Austenite Grain (μm)	Size of Crystallographic Grain (µm)
(a)	91.17	11.51
(b)	98.13	8.30

**Table 6:** Percentage of different grain misorientation angles corresponding to samples shown in Figure 7.

Sample	<3° (%)	>5° (%)	>10° (%)	>15° (%)	>30° (%)	>40° (%)
(a)	56.60	35.17	18.70	15.11	13.91	13.75
(b)	57.06	35.40	22.53	18.20	15.72	15.36

**Table 7:** Results of V-notch Charpy impact tests carried out on simulated CGHAZ at −20 °C (J).

Heat Input kJ/cm	0.073% Nb			0.110% Nb		
	Max	Min	Mean	Max	Min	Mean
20	87	64	79	200	162	182
200	62	19	43	11	8	10

## References

[1] Honeycombe R.W.K., Bhadeshia H.K.D.K., Edward A. (1995). Steel Microstructure and Properties.

[2] Ohkita S., Horii Y. (1995). Recent Development in Controlling the Microstructure and Properties of Low Alloy Steel Weld Metals. ISIJ Int..

[3] Hidesjö C., Svensson L.E. (1999). Consumables for welding of high strength steels. Exploit. Adv. Arc Weld. Technol..

[4] Moon J., Kim S., Jeong H., Lee J., Lee C. (2007). Influence of Nb addition on the particle coarsening and microstructure evolution in a Ti-containing steel weld HAZ. Mate. Sci. Eng. A.

[5] Moon J., Lee J., Lee C. (2007). Prediction for the austenite grain size in the presence of growing particles in the weld HAZ of Ti-microalloyed steel. Mate. Sci. Eng. A.

[6] Miao C., Zhang L.M.S. (2010). Microstructure and toughness of HAZ in X80 pipeline steel with high Nb content. Acta Metall. Sin..

[7] Asselin E., Ahmed T.M., Alfantazi A. (2007). Corrosion of niobium in sulphuric and hydrochloric acid solutions at 75 and 95 °C. Corros. Sci..

[8] Nam N.D., Kim J.C. (2010). Effect of niobium on the corrosion behaviour of low alloy steel in sulfuric acid solution. Corros. Sci..

[9] Tian D. (1998). Microstructure, Cleavage Fracture and Toughness of Granular Bainite in Simulated Coarse-Grained Heat-Affected Zones of Low-Carbon High-Strength Steels. Ph.D. Thesis.

[10] Wu W., Wang Q., Yang L., Liu Z., Li X., Li Y. (2020). Corrosion and SCC initiation behavior of low-alloy high-strength steels microalloyed with Nb and Sb in a simulated polluted marine atmosphere. J. Mater. Res. Technol..

[11] Zhang X., Wei W., Cheng L., Liu J., Wu K., Liu M. (2019). Effects of niobium and rare earth elements on microstructure and initial marine corrosion behavior of low-alloy steels. Appl. Surf. Sci..

[12] Wu W., Liu Z., Wang Q., Li X. (2020). Improving the resistance of high-strength steel to SCC in a SO_2_-polluted marine atmosphere through Nb and Sb microalloying. Corros. Sci..

[13] Yan L.I., Shao M., Zhao Z. (2019). Corrosion resistance of weathering steel containing niobium in marine atmospheric conditions. Corros. Prot..

[14] Lee S., Kim B.C., Kwon D. (1992). Correlation of microstructure and fracture properties in weld heat-affected zones of thermomechanically controlled processed steels. Metall. Trans. A.

[15] Tian D.W., Karjalainen L.P., Qian B.N., Chen X.F. (1996). Correlation between microstructural features of granular bainite, roughness of fracture surface and toughness of simulated CGHAZ in QT type HSLA steels. Scand. J. Metall..

[16] Verrier P., Maurickx T., Taillard R., Garrigues G. (1989). Effect of HAZ microstructure on fracture toughness of offshore microalloyed structural steels. Trid.

[17] Haze T. Influence of toughness and size of local brittle zone on HAZ toughness of HSLA steels. Proceedings of the Proceedings of the 7th International Conference on Offshore Mechanics and Arctic Engineering.

[18] Chijiiwa R., Haze T., Matsuda S., Mimura H., Yamamoto K. (1989). A New Developed Ti-Oxide Bearing Steel Having High HAZ Toughness.

[19] Furuya H., Uemori R., Aihara S., Tomita Y., Hagiwara Y. (2000). Ductile crack propagation characteristics and mechanism of structural steel under high strain rate. ISIS.

[20] Zhao M.C., Yang K., Shan Y.Y. (2002). The effects of thermo-mechanical control process on microstructures and mechanical properties of a commercial pipeline steel. Mate. Sci. Eng. A.

[21] Zhong Y., Xiao F., Zhang J., Shan Y., Wang W., Yang K. (2006). In situ TEM study of the effect of M/A films at grain boundaries on crack propagation in an ultra-fine acicular ferrite pipeline steel. Acta. Mater..

[22] Kumar S., Nath S.K., Kumar V. (2016). Continuous cooling transformation behavior in the weld coarse grained heat affected zone and mechanical properties of Nb-microalloyed and HY85 steels. Mater. Design..

[23] Wang H.H., Qin Z.P., Wan X.L., Wei R., Wu K.M., Misra D. (2017). Continuous cooling transformation behavior and impact toughness in heat-affected zone of Nb-containing fire-resistant steel. Met. Mater. Int..

[24] Li Y., Wan X.L., Lu W.Y., Shirzadi A.A., Isayev O., Hress O., Wu K.M. (2016). Effect of Zr-Ti combined deoxidation on the microstructure and mechanical properties of high-strength low-alloy steels. Mater. Sci. Eng. A.

[25] Sun L., Li H., Zhu L., Liu Y., Hwang J. (2020). Research on the evolution mechanism of pinned particles in welding HAZ of Mg treated shipbuilding steel. Mater. Design..

[26] Liu Y., Li G., Wan X., Zhang X., Shen Y., Wu K. (2017). Toughness improvement by Zr addition in the simulated coarse-grained heat-affected zone of high-strength low-alloy steels. Ironmak. Steelmak..

[27] Wang X., Wang C., Kang J., Yuan G., Misra RD K., Wang G. (2020). An in-situ microscopy study on nucleation and growth of acicular ferrite in Ti-Ca-Zr deoxidized low-carbon steel. Mater. Charact..

[28] Song M.M., Song B., Zhang S.H., Yang Z.B., Xue Z.L., Song S.Q., Xu R.S., Tong Z.B. (2018). Effect of heat input on microstructure and toughness of rare earth-contained C–Mn steel. J. Iron Steel Res. Int..

[29] Mu W., Hedström P., Shibata H., Jönsson P.G., Nakajima K. (2018). High-temperature confocal laser scanning microscopy studies of ferrite formation in inclusion-engineered steels: A review. JOM.

[30] Wang C., Misra RD K., Shi M.H., Zhang P.Y., Wang Z.D., Zhu F.X., Wang G.D. (2014). Transformation behavior of a Ti-Zr deoxidized steel: Microstructure and toughness of simulated coarse grain heat affected zone. Mater. Sci. Eng. A..

[31] Wang C., Wang Z., Wang G. (2016). Effect of hot deformation and controlled cooling process on microstructures of Ti-Zr deoxidized low carbon steel. ISIJ Int..

[32] Pu J., Yu S.F., Li Y.Y. (2017). Effects of Zr-Ti on the microstructure and properties of flux aided backing submerged arc weld metals. J. Alloys Compd..

[33] Yan W., Shan Y.Y., Yang K. (2006). Effect of TiN inclusions on the impact toughness of low-carbon microalloyed steels. Metall. Mater. Trans A.

[34] Moon J., Lee C., Uhm S., Lee J. (2006). Coarsening kinetics of TiN particle in a low alloyed steel in weld HAZ: Considering critical particle size. Acta Mater..

[35] Lee J.L., Pan Y.T. (1995). The formation of intragranular acicular ferrite in simulated heat-affected zone. ISIJ Int..

[36] Wan X.L., Wei R., Wu K.M. (2010). Effect of acicular ferrite formation on grain refinement in the coarse-grained region of heat-affected zone. Mater. Charact..

[37] Ramachandran D.C., Moon J., Lee C.H., Kim S.D., Chung J.H., Biro E., Park Y.D. (2021). Role of bainitic microstructures with M-A constituent on the toughness of an HSLA steel for seismic resistant structural applications. Mater. Sci. Eng. A.

[38] Luo X., Chen X., Wang T., Pan S., Wang Z. (2018). Effect of morphologies of martensite–austenite constituents on impact toughness in intercritically reheated coarse-grained heat-affected zone of HSLA steel. Mater. Sci. Eng. A.

[39] Huda N., Midawi A., Gianetto J.A., Gerlich A.P. (2021). Continuous cooling transformation behaviour and toughness of heat-affected zones in an X80 line pipe steel. J. Mater. Res. Technol..

[40] Wang C., Wu X., Liu J. (2006). Transmission electron microscopy of martensite/austenite islands in pipeline steel X70. Mater. Sci. Eng. A.

[41] Matsuda F., Ikeuchi K., Okada H., Hrivnak I., Park H.S. (1994). Effect of MA Constituent on Fracture Behavior of 780 and 980MPa Class HSLA Steels Subjected to Weld HAZ Thermal Cycles (Materials, Metallurgy& Weldability). Trans. JWRI.

[42] Kang S., Speer J.G., Regier R.W., Nako H., Kennett S.C., Findley K.O. (2016). The analysis of bainitic ferrite microstructure in microalloyed plate steels through quantitative characterization of intervariant boundaries. Mater. Sci. Eng. A.

[43] Gan X., Wan X., Zhang Y., Wang H., Li G., Xu G., Wu K. (2019). Investigation of characteristic and evolution of fine-grained bainitic microstructure in the coarse-grained heat-affected zone of super-high strength steel for offshore structure. Mater. Charact..

[44] Nasiri Z., Ghaemifar S., Naghizadeh M., Mirzadeh H. (2021). Thermal mechanisms of grain refinement in steels: A review. Met. Mater. Int..

